# *In vivo* modelling of cutaneous T-cell lymphoma: The role of SOCS1

**DOI:** 10.3389/fonc.2022.1031052

**Published:** 2022-11-24

**Authors:** Yixin Luo, Maarten H. Vermeer, Frank R. de Gruijl, Willem H. Zoutman, Marjolein Sluijter, Thorbald van Hall, Cornelis P. Tensen

**Affiliations:** ^1^ Department of Dermatology, Leiden University Medical Center, Leiden, Netherlands; ^2^ Department of Medical Oncology, Oncode Institute, Leiden University Medical Center, Leiden, Netherlands

**Keywords:** cutaneous T cell lymphoma, mycosis fungoides, transgenic mouse, *in vivo* modeling, inflammation

## Abstract

**Introduction:**

Mycosis fungoides (MF), the most common type of Cutaneous T cell Lymphoma (CTCL), is characterized by an inflamed skin intermixed with proliferating malignant mature skin-homing CD4+ T cells. Detailed genomic analyses of MF skin biopsies revealed several candidate genes possibly involved in genesis of these tumors and/or potential targets for therapy. These studies showed, in addition to common loss of cell cycle regulator CDKN2A, activation of several oncogenic pathways, most prominently and consistently involving JAK/STAT signaling. SOCS1, an endogenous inhibitor of the JAK/STAT signaling pathway, was identified as a recurrently deleted gene in MF, already occurring in the earliest stages of the disease.

**Methods:**

To explore the mechanisms of MF, we create *in vivo* mouse models of autochthonous CTCLs and these genetically engineered mouse models (GEMMS) can also serve as valid experimental models for targeted therapy. We describe the impact of allelic deletion of *Socs1* in CD4 T cells of the skin. To achieve this, we crossed inducible Cre-transgenic mice in the CD4 lineage with transgenic mice carrying floxed genes of *Socs1*. We first determined optimal conditions for *Socs1* ablation with limited effects on circulating CD4 T-cells in blood. Next, we started time-course experiments mimicking sustained inflammation, typical in CTCL. FACS analysis of the blood was done every week. Skin biopsies were analyzed by immunocytochemical staining at the end of the experiment.

**Results:**

We found that the *Socs1* knockout transgenic group had thicker epidermis of treated skin compared with the control group and had more CD3 and CD4 in the skin of the transgenic group compared to the control group. We also noted more activation of *Stat3* by staining for P-Stat3 in *Socs1* knockout compared to wt CD4+T cells in the skin. The results also indicated that single copy loss of *Socs1* in combination with sustained inflammation is insufficient to start a phenotype resembling early stage mycosis fungoides within eight weeks in these mice.

**Conclusion:**

In sum, we developed and optimized an autochthonous murine model permitting selective knockout of *Socs1* in skin infiltrating CD4 T-cells. This paves the way for more elaborate experiments to gain insight in the oncogenesis of CTCL.

## Introduction

Mycosis fungoides (MF), the most common type of Cutaneous T cell Lymphoma (CTCL), is characterized by an inflamed skin intermixed with proliferating malignant mature skin-homing CD4+ T cells (1–3). It presents in the early stage with cutaneous patches and/or plaques. The disease has a favorable prognosis in those early stages (IA-IB). However, approximately 25% of patients progress to the advanced stage, presented with cutaneous tumors or erythroderma or systemic involvement, and a dramatic reduction in five-year survival from approximately 80% to 25%.

The exact molecular mechanisms of MF pathology remained unclear despite some genomic and gene expression profile studies. Recent detailed genomic analyses (using next-generation sequencing) of MF skin biopsies revealed several candidate genes possibly involved in the genesis of these tumors and/or potential targets for therapy (4). These studies showed, in addition to the common loss of cell cycle regulator CDKN2A, activation of several oncogenic pathways, most prominently and consistently involving JAK/STAT signaling. However, the precise genetic alterations driving these oncogenic pathways, the genetic drivers, remained unclear.

In mycosis fungoides, SOCS1 was identified as one of the highly recurrently deleted tumor suppressors and the gene rearrangements of SOCS1 were already present in the earliest stages of MF (5). SOCS1 belongs to the suppressor of cytokine signaling (SOCS) family and is an endogenous inhibitor of the JAK/STAT signaling pathway, inhibiting JAK/STAT phosphorylation and activation *via* a negative feedback loop (6). It plays critical roles in Th subset differentiation (7). and the regulation of Tregs (8). SOCS1 is a unique tumor-suppressor gene that regulates inflammation-related tumorigenesis (9).

In early disease stages, the skin lesions contain of a small population of malignant T cells immersed within a dense infiltrate of reactive immune cells. It is supposed that chronic inflammation precedes and gives rise to the malignant cell clone, which takes the upper hand as the tumor progresses (10). It is now recognized that inflammation may not only combat the tumor but may promote its development. Immunologic processes, and in particular chronic inflammation, were added to Weinberg’s and Hanahan’s original hallmarks of cancer (11).

To elaborate on the function of SOCS1 and other identified genes in mycosis fungoides, in particular in the initiating events, we aim to use mouse models. The currently available mouse models (12) are nearly all based on cell lines and xenografts in immune compromised mice and models that represent early stages of MF are lacking. Here we describe the development of a genetically engineered mouse model that aims to represent autochthonous CTCLs, permitting the necessary next steps in dissecting the precise role(s) of identified genes in the pathogenesis starting with SOCS1. We show that Socs1 deletion in this *in vivo* model is limited to CD4 T cells and chronic inflammation of the skin can be maintained and eventually used to promote and enhance the tumorigenic process. The irreplaceable merit of this autochthonous model is the possibility to study in detail the impact of the interaction between the imposed tumor cells transformation and an intact immune system. Finally, genetic mouse models might also serve as valid experimental models for targeted therapy.

## Materials and methods

### Mice

Conditional *Socs1* knockout mice (13) (floxed *Socs1*) and tamoxifen-inducible Cd4-driven CreERT2-knock-in mice (Cd4Cre) (14) were crossed. The Cd4-driven CreERT2-knock-in mice were purchased from Jackson’s Laboratories (#:022356). Conditional *Socs1* knockout mice (with loxP sites on either side of exon 2 of the targeted *Socs1* gene; with inserted reporter human CD4) were kindly obtained from Professor Warren Alexander at Walter and Eliza Hall Institute. The first round of crossing yielded Socs1 fl/wt Cd4Cre+/- and Socs1 fl/wt Cd4Cre-/- mice. The resulting offspring will have exon2 of *Socs1* deleted in Cre-expressing CD4 T cells up on administering tamoxifen.

Of note is the sub-Mendelian low yield of Socs1 fl/fl Cd4Cre pups which hampered populating the experiments with an adequate number of these mice.

Genomic PCR was conducted to analyze the genotypes of mice using ear DNA and gene-specific primers (**Table S1**) for the Socs1flox transgene and Cd4Cre construct.

All mice were housed in individually ventilated cages, maintained under specific pathogen-free conditions, and had access to food and water ad libitum.

All mouse experiments were supervised by the animal welfare committee (IvD) of the Leiden University Medical Center and approved by the national central committee of animal experiments (CCD) under the permit number AVD116002015271, in accordance with the Dutch Act on Animal Experimentation and EU Directive 2020/63/EU.

Mice entered the experiments at ages between 6 and 20 weeks. The mice were assigned to control or experimental groups based on genotype and were assigned randomly to experimental treatments within each group.

### Preparation and administration of oxazolone and 4-hydroxy-tamoxifen

Oxazolone (4-Ethoxymethylene-2-phenyl-2-oxazolin-5-one, Sigma-Aldrich, Netherlands) was dissolved in acetone. For every experiment performed, a freshly made solution was used. Mice were sensitized with 1.5% oxazolone (100*μl*) on the shaved abdomen skin (2cm × 2cm) under anesthesia. After seven days, mice were challenged with 0.5% oxazolone (150 *μl*) on the shaved left flank skin (2cm × 3cm) and vehicle only (150 *μl* acetone) on the shaved right flank skin (2cm × 3cm). To maintain the skin inflammation, mice received 0.5% oxazolone (150 *μl*) on the shaved left flank skin (2cm × 3cm) and again vehicle only (150 *μl* acetone) on the shaved right flank skin (2cm × 3cm) three times a week.

4-hydroxy-Tamoxifen (4OHT, Sigma-Aldrich, Netherlands) was dissolved in ethanol (20mg/ml) and was sonicated for 2 minutes. Then it was stored at -20 °C for the experiments. For topical administration, 4OHT was reheated at 60 °C for 10 minutes and was administered 1mg per mouse topically on left shaved skin (2cm × 3cm).

### Flow cytometry

Blood (50 *μl*) was drawn from the tail veil every week. This was performed at least 24 hours after OXA application. Whole blood samples were processed using lysis buffer (from Hospital Pharmacy at LUMC) for 10 mins at 37°C. Cells were incubated with monoclonal antibodies for 30 min on ice.

Fluorescence-labeled antibodies including anti-mouse CD3 (clone 145-2C11, BD, The Netherlands), anti-mouse CD19(clone 1D3, Thermo Fisher Scientific, The Netherlands), anti-mosue CD4 (clone RM4-5, Thermo Fisher Scientific, The Netherlands), anti-mouse CD8 (clone 53-6.7, Biolegend, The Netherlands) and anti-ΔhCD4 (clone RPA-T4, eBioscience™, The Netherlands). Of note, the antibody for ΔhCD4 should be specifical clone that fits for the surrogated reporter in Socs1flox transgenic mouse. Samples were processed in a BD Fortessa flow cytometer and analyzed using the FlowJo software.

### Histological and immunohistochemical analyses

Skin samples were fixed with 10% neutral buffered formalin, dehydrated with increasing grades of ethanol, cleared with xylene, and embedded in paraffin. Sections (4 *μ*m-thick) were cut with a microtome (Leica 149MULTI0C1). Tissue sections were stained with hematoxylin and eosin to visualize general histological architecture.

For immunohistochemical analyses, paraffin-embedded skin sections were dewaxed with xylene and rehydrated. After that, the sections were blocked for endogenous peroxidase using 0.3% hydrogen peroxide and nonspecific antibody binding using a blocking buffer (SuperBlock, Thermo Fisher Scientific, The Netherlands). Antigen retrieval was performed using citric acid (PH6.0) solution. The tissue sections were incubated with the following primary antibodies at 4°C overnight: anti-human CD4 (1:2000, EPR6855, Abcam, The Netherlands), anti-mouse CD3 (1: 200, D7A6E, Cell Signaling Technology, The Netherlands), anti-mouse CD4 (1:100, D7D2Z, Cell Signaling Technology, The Netherlands), anti-mouse CD8 (1:1600, 4SM15, eBioscience™, The Netherlands), anti-phospho-Stat3 (1:150, D3A7, Cell Signaling Technology, The Netherlands).

Then sections were incubated with secondary antibody at room temperature for 60 min. Sections were visualized with Vectastain Elite Kit (Vector Labs, Netherlands) and diaminobenzidine (Dako Omnis, Agilent Dako, Netherlands). After counterstaining with hematoxylin, sections were mounted. The scanner (3DHISTECH, Pannoramic 250) was used for microscopic examination and image acquisition.

### Immunohistochemical evaluation

The layers of the epidermis were counted within at least 5 high power fields (HPF) (20x magnification) of each slide, and the means were assessed for further statistical analysis.

The numbers of △hCD4+, CD3+, CD4+, CD8+ and phospho-Stat3 positive cells in the dermis were counted within at least 5 HPF (20x magnification) per case. The values were normalized to cells/mm^2^, and the mean numbers were assessed for further statistical analysis. The evaluations were conducted by two independent individuals who were blinded to samples information.

### Statistical analysis

A paired t-test was used to compare treated skin and untreated skin from the same mouse group. Nonparametric test and the analysis of covariance were used to compare the skin between two different mouse groups.

All statistical analyses were performed using GraphPad Prism software version 8 (GraphPad). In all cases a P-value of 0.05 and below was considered significant (*), P < 0.01(**) and P < 0.001 (***) as highly significant.

## Results

### Generation of a specific conditional *Socs1* knockout mouse model

**Figure 1:**
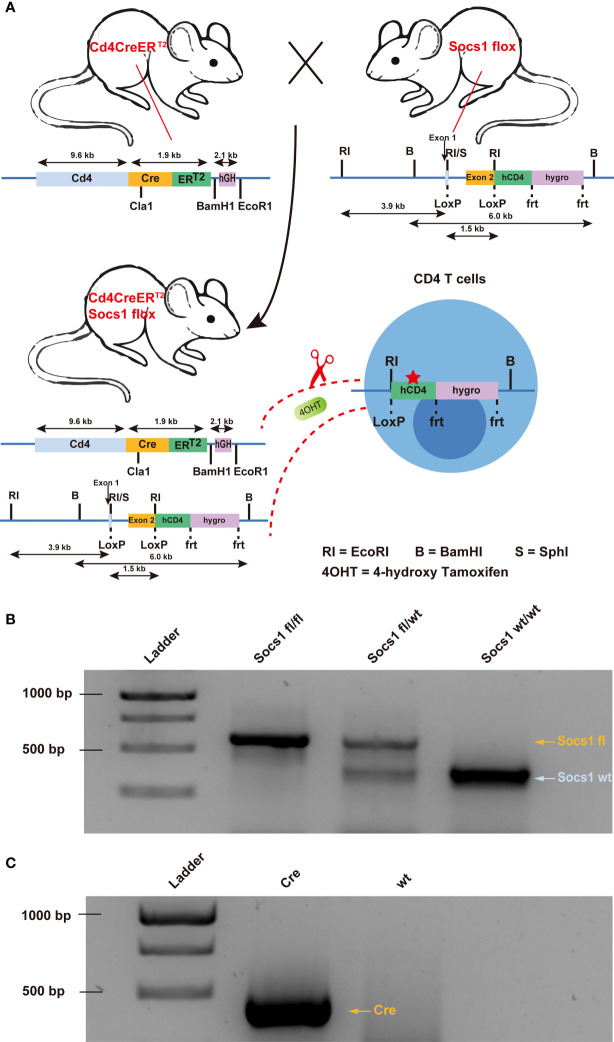
Creation of conditional *Socs1* knockout mice permitting specific inactivation of the *Socs1* gene in CD4 T cells. **(A)**. Breeding scheme used to create on conditional knockout mice permitting specific deletion of functional Socs1 in CD4 T cells. The transgenic Socs1flox Cd4Cre+ (Socs1fl/wt Cd4Cre+ and Socs1fl/fl Cd4Cre+) mice were generated by crossing Socs1flox (in which the exon2 of *Socs1* is flanked by LoxP sites) with the inducible Cd4CreERT2 mice (in which Cre is under the control of CD4 promoter. Expression of the Cre recombinase gene is induced in CD4 T cells by giving the mice the drug tamoxifen, (shown in the figure as 4OHT). Tamoxifen allows the Cre recombinase to enter the nucleus of CD4 T cells and recombine the loxP sites. In the event that Socs1fl was deleted by Cre, the inserted reporter human CD4 (hCD4) will be under control of the endogenous *Socs1* promoter and expressed instead of *Socs1*. The human CD4 reporter contains a F43I mutation and intracellular truncation to abrogates its function. **(B)**. Agarose gel electrophoresis image of the *Socs1* PCR product of Socs1fl/fl, Socs1fl/wt and Socs1wt/wt mice. Visible marker bands indicate fragment sizes of 1000, 750, 500 and 250 bps from top to bottom (lane 1). **(C)**. Agarose gel electrophoresis image of the Cre PCR product of the Cd4Cre+ and wildtype mice. Visible marker bands indicate fragment sizes of 1000, 750 and 500 bps from top to bottom (lane 1).

To knock out the *Socs1* gene in murine CD4 (mCD4) T cells, we crossed conditional *Socs1* knockout mice (floxed *Socs1*) and tamoxifen-inducible Cd4-driven CreERT2-knock-in mice (Cd4Cre). The tamoxifen-inducible Cd4-driven CreERT2 transgenic mouse strain expresses a tamoxifen inducible Cre recombinase (CreERT2) under the control of the *Cd4* gene promoter (**Figure 1A**). In the conditional *Socs1* knockout mouse, the endogenous *Socs1* gene was replaced with a modified *Socs1* gene flanked by LoxP sites. The modified *Socs1* gene harbors a 3’ reporter, △hCD4, which comes under the control of the *Socs1* promoter. This reporter, ‘△ human CD4’ contains an F43I mutation and intracellular truncation, which abrogates its function.

In Socs1flox Cd4Cre+/- mice (Socs1 fl/wt Cd4Cre+/- and Socs1 fl/fl Cd4Cre+/-), Cre recombinase is activated selectively in CD4 T cells upon tamoxifen and deletes the *Socs1* sequence between loxP sites. *Socs1* wild-type alleles, *Socs1* floxed alleles and Cd4CreERT2 transgene were determined by PCR using genomic DNA from ear clips. (**Figures 1B, C**).

### Three doses of 4OHT topical application have less systemic influence compared with five doses of 4OHT topical application

**Figure 2:**
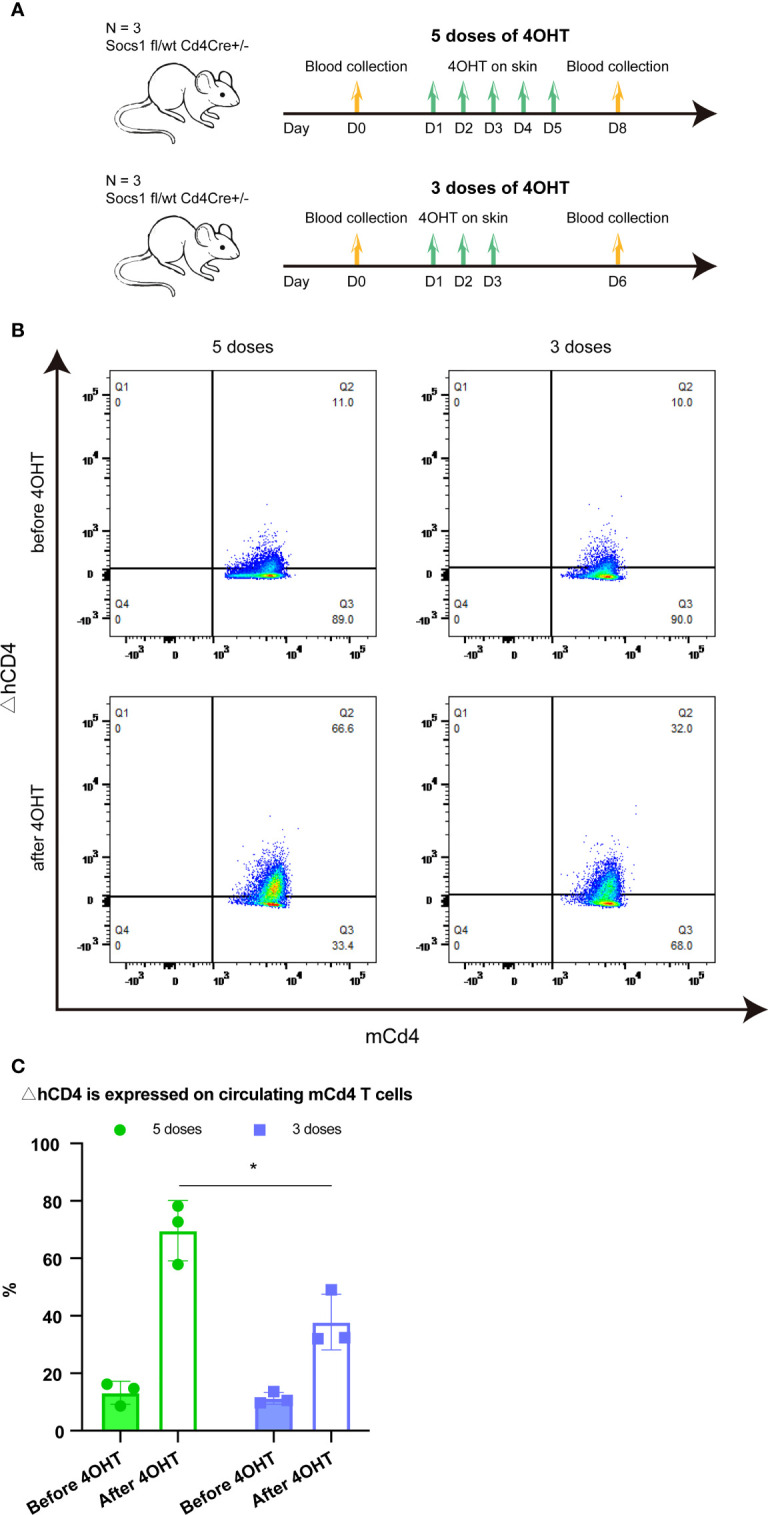
Effect of five and three times 4OHT application on the skin **(A)**. Schematic representation of the experiment determining the effect of 5 and 3 doses of 4OHT. Blood (50μl) was drawn from the tail veil before 4OHT application and 3 days after the last 4OHT application and subjected to FACS analysis. **(B)**. Reporter ΔhCD4 expression in circulating mCd4 T cells using FACS analysis. Representative flow plots of blood obtained before 5 doses of 4OHT, after 5 doses of 4OHT, before 3 doses of 4OHT, and after 3 doses of 4OHT. **(C)**. Cells with Socs1 deletion (resulting in ΔhCD4 expression) in percentage of total circulating murine CD4 T cells in transgenic Socs1fl/wt Cd4Cre+ mice. Data are presented as mean ± SD. Symbols in bar graphs represent individual mouse. *P<0.05.

Two groups of Socs1 fl/wt Cd4Cre+/- mice, with bilateral flank skin shaved, received topical 4OHT once daily on the left flank and acetone as a vehicle control once daily on the right flank for 5 and 3 consecutive days, respectively. Blood (50ul) was taken from the tail vein before the 4OHT application and three days after the last 4OHT application. (**Figure 2A**)

The *Socs1* deletion, showed by the reporter gene △hCD4 on mCD4 T cells, was measured by flow cytometry in both groups. The results showed that *Socs1* was successfully deleted in circulating mCD4 T cells after the 4OHT application in both groups of mice. Moreover, it confirmed that the activation of Cre-loxP system in our specific mouse model could be achieved by using five doses of 4OHT and three doses of 4OHT, which resulted in the deletion of *Socs1* in mCD4T cells. (**Figure 2B**) After 4OHT, the Socs1 fl/wt Cd4Cre+/- mouse can be marked as the Socs1-/wt Cd4Cre+/- (S+-C) mouse.

In quantifying the percentage of mCD4 T cells with *Socs1* deletion in circulating mCD4 T cells after 5 and 3 doses of 4OHT, we observed a clear dose effect: 3 doses resulted in statistically significantly less deletions than 5 doses. (**Figure 2C**)

### Long-term low concentration OXA is suitable for inducing and maintaining an inflamed skin

**Figure 3:**
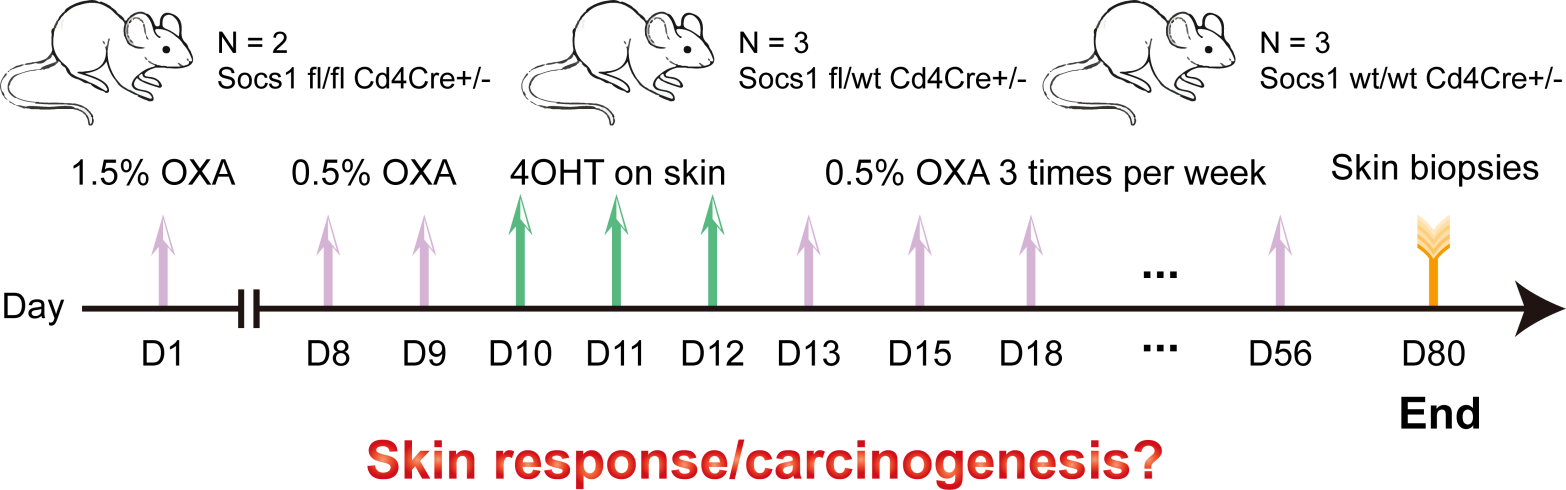
Scheme of the experiment performed using the conditional *Socs1* knockout mouse model. OXA also on the skin.

In this experiment, we tried to avoid the damaging effect on murine skin of a high concentration of OXA thereby reducing mouse distress/scratching, and optimized the OXA dose to induce a sustainable skin inflammation in skin (no wounding from scratching). 1.5% OXA was used for sensitization on the shaved abdomen on day 1. 0.5% OXA was used for challenge on the shaved left flank seven days later. Then 4OHT on the shaved left flank was used for three consecutive days to knockout *Socs1*. After that, repeated dosing was performed on the shaved left flank until day 56, with each dose at least 48h apart. Skin samples were collected two weeks after the last dose. (**Figure 3**)

After 4OHT, Socs1fl/fl Cd4Cre+/- mouse are abbreviated as S–C (Socs1-/- Cd4Cre+/-) mouse, and the Socs1wt/wt Cd4Cre+/- mouse as the C (control) mouse.

During the study, none of the mice showed open wounds or persistent severe pruritus on the skin. The treated skin had obvious inflammation symptoms like erythema, scaling, and skin roughness. The S–C group had the strongest skin inflammation among three groups. Meanwhile, the shaved right flank with vehicle had no inflammation phenotype in each group of mice (**Figure S1**). Flow cytometry data from weekly peripheral blood during the experiment also showed no significant abnormalities in the immune system of the mice.

### *Socs1* was successfully deleted in circulating and skin-homing CD4 T cells of the transgenic mouse.

**Figure 4:**
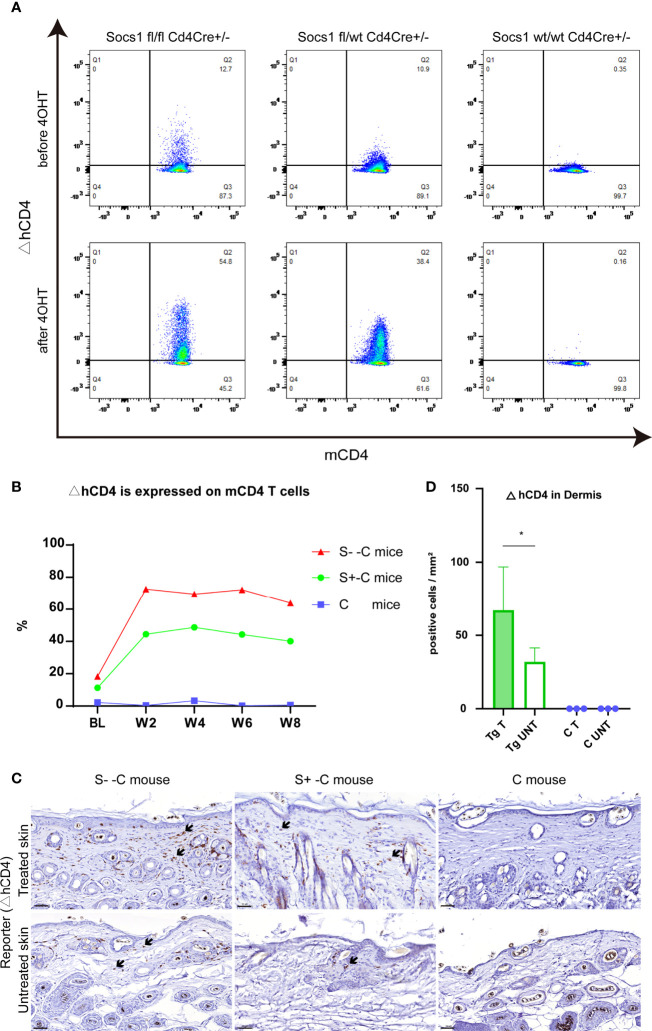
*Socs1* can successfully be deleted in circulating and skin-homing CD4 T cells of the transgenic mouse by 4OH Tamoxifen application. **(A)**. Circulating murine Cd4 T cells were analyzed for ΔhCD4 expression by flow cytometry to show *Socs1* deletion. Representative flow cytometry plots for before and after 4OHT application in Socs1fl/fl mice Cd4Cre+/-, Socs1fl/wt Cd4Cre+/- mice and Socs1wt/wt Cd4Cre+/- mice. **(B)**. *Socs1* deletion (measured as ΔhCD4 expression) in cells as a percentage of total circulating murine CD4 T cells in S–C mice, S+-C mice and C mice during the whole experiment. Each plot is the mean value of mice with the same genotype. BL is baseline. W is week. S–C is Socs1 -/- Cd4Cre+/-; S+-C is Socs1-/wt Cd4Cre+/-; C is control group. **(C)**. Immunohistochemical staining of ΔhCD4 to show *Socs1* deletion in cells in the murine skin. Representative photomicrographs of ΔhCD4 expression in treated and untreated skin from S–C mice, S+-C mice and C mice respectively. Black arrows: ΔhCD4 positive cells. Scale bar: 50 μm. S–C is Socs1 -/- Cd4Cre+/-; S+-C is Socs1-/wt Cd4Cre+/-; C is control group. **(D)**. Quantification of ΔhCD4 positive cells in dermis of transgenic group (S–C mice, S+-C mice) and C mice. Data are presented as mean ± SD (N= 5 in Tg and N=3 in C). *P<0.05. Tg is transgenic group, C is control group. S–C is Socs1 -/- Cd4Cre+/-; S+-C is Socs1-/wt Cd4Cre+/-.

To confirm the *Socs1* deletion in circulating CD4 T cells, we measured the reporter △hCD4 in peripheral blood by flow cytometry. The results showed that *Socs1* was deleted in circulating mCD4 T cells in S–C and S+-C groups after the 4OHT application on the skin. In C group, no *Socs1* deletion was detected. (**Figure 4A**) The *Socs1* deletion level in circulating mCD4 T cells from S–C and S+-C groups was long-lasting during the experiment (**Figure 4B**). It illustrates the stable knockout effect of our new transgenic mouse strain.

The *Socs1* deletion in skin resident CD4 T cells, showed by reporter gene △hCD4, was confirmed by immunohistochemical staining. The results showed that there were △hCD4 positive cells in S–C and S+-C groups after the 4OHT application on the skin. The number of △hCD4 positive cells was most pronounced in the S–C group although this was not firmly quantifiable with only two mice in this genotype group (see M&M section). In the control group, no *Socs1* deletion was detected. (**Figure 4C**) Among transgenic mice (S- -C and S+- C), there is statistically significant more *Socs1* knockout in the dermis of the treated skin comparison with that of the untreated skin. (**Figure 4D**)

### Augmented inflammation in *Socs1* knockout transgenic mouse

**Figure 5:**
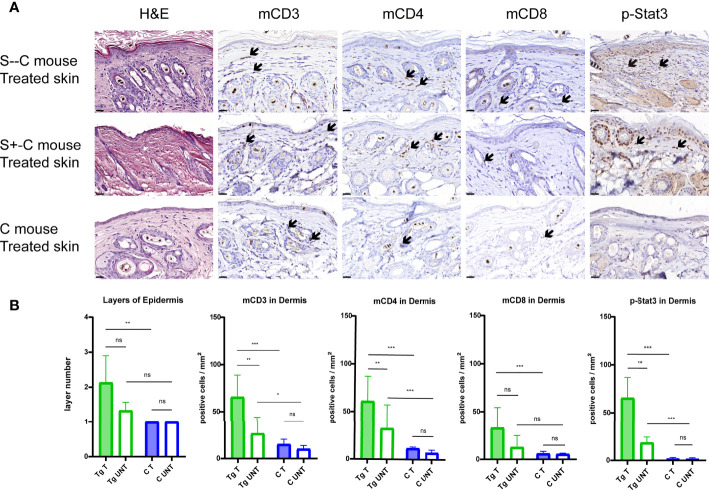
Detailed histological analyses using H&E and immunohistochemical staining of murine skin sections. **(A)**. Representative photomicrographs of treated skin from S–C mice, S+-C mice and C mice respectively. Black arrows: ΔhCD4 positive cells. Scale bar: 50 μm. S–C is Socs1 -/- Cd4Cre+/-; S+-C is Socs1-/wt Cd4Cre+/-; C is control group. **(B)**. Quantification of the layers of epidermis, inflammatory cells and pStat3 positive cells in the dermis from transgenic group (S–C mice, S+-C mice) and C mice. Data are presented as mean ± SD. (N=5 and 3 in each group). *P<0.05, **P<0.01, ***P<0.001. ns is no significance. Tg is transgenic group, C is control group. S–C is Socs1 -/- Cd4Cre+/-; S+-C is Socs1-/wt Cd4Cre+/-.

We performed immuno-histopathology on skin biopsies to characterize the effect of *Socs1* deletion in our transgenic mice with chronic skin inflammation. The H&E staining of the skin sections from the treated flanks of three group mice showed the thickness of the epidermis of treated skin was most pronounced in the S–C group although this could not be well evaluated statistically with only two mice. (**Figure 5A**) The epidermal layers were assessed and quantitated in the transgenic group (S- -C and S+-C) and the C groups. The transgenic group had thicker epidermis of treated skin compared with the control group. (**Figure 5B**)

The inflammatory response was confirmed by immunohistochemical staining of mCD3, mCD4 and mCD8 of the skin sections of the mice. The numbers of inflammatory cells were most pronounced in the S–C group although this was again not well quantitated with only two mice in this genotype group. Quantifying positive staining cells showed a statistically significant increase in mCD3, mCD4 and mCD8 cells in the dermis of the treated skin of mice in the transgenic group (S- -C and S+-C) in comparison with the C group. In the untreated skin dermis, the numbers of mCD3 and mCD4 in the transgenic group (S- - C and S+-C) also showed a statistically significant increase comparison with the C group. The numbers of mCD8 in the untreated skin dermis of mice in the transgenic group (S- - C and S+-C) was not different from that in the untreated skin dermis of mice in the C group. (**Figure 5B**)

In the transgenic group (S- - C and S+-C), there was statistically significantly more mCD3 and mCD4 staining in the dermis of the treated flank than that of untreated skin. For mCD8, the dermis of treated and untreated flank was not different. Moreover, there was no difference in inflammatory cells in the treated and non-treated skin of the control group. The mCD3 and mCD4 were more abundant in the skin of the transgenic group compared to the control group, suggesting that *Socs1* deletion can promote skin inflammation. (**Figure 5B**)

### phospho-Stat3 (pStat3) expression in CD4 T cells of transgenic *Socs1* knockout mice

To further characterize the role of SOCS1 in the JAK/STAT signaling pathway, we examined the pStat3 expression in skin biopsies by immunohistochemical staining. *Stat3* activation was confirmed by immunohistochemical staining of pStat3 of the skin sections of the three groups of mice. (**Figure 5A**) The number of pStat3 positive cells was most pronounced in S–C mice although, again, this could not be well statistically evaluated with only two mice in this genotype group. Quantifying positive staining cells showed a statistically significant increase in number of p-Stat3 cells in the dermis of treated skin of mice in the transgenic group (S–C and S+-C) compared to the treated skin in C group. The amount of pStat3 positive cells in the untreated skin dermis of mice in the transgenic group (S–C and S+-C) also showed a statistically significant increase comparison with the C group. (**Figure 5B**)

In the transgenic group (S–C and S+-C), there were statistically significantly more pStat3 positive cells in the dermis of the treated flank compared to untreated skin. In contrast, there was remarkably no difference in pStat3 positive cells in the treated and non-treated skin of the Control group. (**Figure 5B**)

## Discussion

This study is, to our best knowledge, the first that specifically focused on the function of genes involved in mycosis fungoides by targeted deletion in skin homing T-cells in combination with chronic inflammation using GEMMs.

To overcome the prenatal lethality of *Socs1* deficiency during development (13, 15, 16) and frame the *Socs1* deletion in CD4 T cells specifically, thus enabling studies on the contribution of *Socs1* in the pathogenesis of mycosis fungoides, we used cross breeding of two existing transgenic mouse strains (Socs1flox and Cd4Cre). This resulted in the expected genotypes, however, a sub-Mendelian low yield of Socs1 fl/fl Cd4Cre pups was observed probably due to leakage of Cre.

We first show that application of 4OH tamoxifen on the skin of these transgenes effectively targets local Cd4-Cre T-cells and that a decrease from five to three 4OHT applications statistically significantly reduces the systemic effect on circulating T cells. There are several ways to apply Tamoxifen to activate Cre-loxP systems depending on various research aims (17, 18). A majority of studies use intraperitoneal injections as well as oral gavage to get the potent systemic effect and the commonly used dosing is usually on 5 consecutive days (Tamoxifen 1mg/per day/per mouse, 4OHT 1mg/per day/per mouse) (19, 20). Since our study’s target tissue are immune cells in the skin, we reasoned that topical administration would be preferable. Five doses and three doses of 4OHT topical application have been applied in a few studies all targeting keratinocytes while studies that distinguish the systemic effect from topical application are not available (21–23). This study shows that Cre can be activated to knock out *Socs1* in skin resident T-cells in a way that minimized the systemic effect of 4OHT on other circulating immune cells animals.

Next we determined optimal conditions for simulation of a chronic dermatosis without damaging skin integrity. Inflammation can exhibit tumor-promoting effects even at the early phases of neoplastic growth and is characteristic for early stage mycosis fungoides (11, 24). In addition, it can promote the progression of precancerous neoplasia into full-blown malignancies (25–28). In our study we induced chronic skin inflammation using Oxazolone to add the possible tumor-promoting effects in the transgenic mouse, but also to attract T-cells to the skin before application of 4OHT to increase the probability to hit the target cells. In previous studies, mouse ears were mostly used as a model for OXA-induced long-term chronic skin inflammation (29–32). Commonly used concentrations of oxazolone are 1.5% - 2% in the sensitization phase and 0.4-1% in the challenging phase. The maximum duration of repeated use of OXA for long-term chronic inflammation studies was 3 weeks (29, 33). In our study, low concentrations of OXA were repeatedly applied 3 times per week on the flank of mice until 8 weeks (Day 56). The mice showed typical inflammatory reactions such as erythema, scaling, and skin roughness. However, open wounds, ulcers, and long-lasting, intense scratching were successfully avoided. It is important in this study not only to exclude a specific tumor promotion from severe skin trauma but also for the well-being of the experimental animals (24).

Our immune-histochemical analyses additionally confirmed *Socs1* was deleted successfully in CD4 T cells in the skin of transgenic mice upon treatment with 4OHT. Expression of the ΔhCD4 reporter (surrogate marker for *Socs1* deletion) in CD4 T cells was primarily observed in the left, treated flank of transgenic Socs1 fl/wt Cd4Cre+/- mice.

The thickness of epidermis and the immune-histochemistry results of mCD3, mCD4 and mCD8 confirmed that *Socs1* deletion augment the skin inflammation in the dermis of Socs1 flox Cd4Cre transgenic mouse specifically. Moreover, the mCD3 and mCD4 infiltration in the dermis of treated flanks are statistically higher than that of untreated flanks. We collected the skin samples two weeks after the last OXA administration to assess prolonged inflammation. The clinically observable skin inflammation in our mouse model was alleviated over this time interval but could still be observed. Histology results showed that the inflammatory response in the treated skin of *Socs1*-deleted mouse group was still obvious. *Socs1* deletion in CD4 T cells increases prolonged skin inflammation. Consistent with our observation, previous research indicates *Socs1* loss relates to inflammation-associated tumor development. The germline loss-of-function mutations in the SOCS1 gene were associated with early onset autoimmune manifestations in a whole exome/genome sequencing study (34). *Socs1* deletion in murine dendritic cells from induce stronger immune responses both *in vitro* and *in vivo* (35).

Finally, we determined whether Jak/Stat signaling was enhanced as a result of *Socs1* deletion by measuring pStat3. Our results indicated more activation of *Stat3* in *Socs1* knockout compared to wt CD4+T cells in the dermis.

These results are in line with the previous findings showing that the phosphorylation of Stat3 was elevated in splenic T cells of *Socs1*-deficient mice relative to those of wild-type mice (36). Downregulation of *Socs1* in rat hepatocytes activates *Jak2-Stat3* in an animal model of sepsis (37). Aberrant activation of the JAK/STAT3 pathway due to down-regulation of SOCS1 by miR-155 is observed in solid tumors, such as breast cancer and laryngeal carcinoma (38, 39). In melanoma, melanoma cell-secreted exosomal miR-155 suppressed SOCS1 expression in CAFs. Suppression of SOCS1 in CAFs activated the JAK2/STAT3 signaling pathway (40). In CTCL, especially in advanced stages, constitutive expression of STAT3 has been consistently observed. IL-21 leads to more specific activation of STAT3 in Sezary Syndrome, which in turn directly upregulates IL-21 expression leading to an enhanced IL-21 autocrine signaling loop (41). Constitutively active STAT3 can increase survival and resistance to apoptosis in malignant T cells by promoting bcl-2 expression (42).

In contrast to tumors, reduced miR-155 expression, upregulated SOCS1 expression, and significantly reduced STAT3 phosphorylation were found in CD4+ T cells in autoimmune SLE. IL21 expression was upregulated but induced STAT3 phosphorylation was inhibited. STAT3 phosphorylation was increased after miR-155 overexpression (43). SOCS1 is necessary for stability and suppressor functions of Treg cells: SOCS1 protects Treg cells from harmful effects of inflammatory cytokines. STAT3 overactivation in Socs1-deficient Treg cells promotes the conversion of Treg cells to Th17-like cells (8). Based on these and other data (4, 5), a model explaining the role of SOCS1 in aberrant JAK/STAT signaling in MF was postulated.

We also observed keratinocytes with more activated Stat3 in the epidermis of Socs1 knockout transgenic mice, probably caused by cytokine production. Stat3 activation is associated with aberrant keratinocytes differentiation and hyperproliferation (44). Stat3 plays a major role in epithelial carcinogenesis. This has been demonstrated in previous studies on wounds healing, UVB-induced skin carcinogenesis and keratinocytes-specific Stat3-deficient mice (45, 46).

There are some limitations in this study. The first is that there are obvious inflammatory responses and Stat3 activation. However, this model has not yet shown clear signs of lymphoma development. The second one is the small size of samples. In the future, we will use the established model to conduct studies with larger sample sizes and that run through a longer period of time to induce enhance the possibility of lymphoma development.

In sum, we developed and optimized an autochthonous murine model permitting selective knockout of *Socs1* in skin infiltrating CD4 T-cells. Our results show that *Socs1* deletion specifically in CD4 T cells can promote persisting inflammation in the skin of mice and activate Stat3. This paves the way for more elaborate experiments, e.g. extending the time of treatment, knockout of other genes to gain insight in the genesis of CTCL.

## Data availability statement

The raw data supporting the conclusions of this article will be made available by the authors, without undue reservation.

## Ethics statement

The animal study was reviewed and approved by The Netherlands National central committee of animal experiments.

## Author contributions

Conceptualization: CT and MV. Methodology: YL, TH, WZ and MS. Data curation: YL, CT and TH. Formal analysis: YL, FG and CT. Writing- original draft: YL. Writing- review and editing: CT, FG and TH. All authors contributed to the article and approved the submitted version.

## Funding

This research was funded by the Chinese Scholarship Council.

## Acknowledgments

We want to thank Professor Warren Alexander at Walter and Eliza Hall Institute for kindly providing the Socs1fl/fl mice. We would like to thank Erno Vreugdenhil for the helpful discussion and Amelia Maduro for helping with photos acquisition and optimization of IHC protocols.

## Conflict of interest

The authors declare that the research was conducted in the absence of any commercial or financial relationships that could be construed as a potential conflict of interest.

## Publisher’s note

All claims expressed in this article are solely those of the authors and do not necessarily represent those of their affiliated organizations, or those of the publisher, the editors and the reviewers. Any product that may be evaluated in this article, or claim that may be made by its manufacturer, is not guaranteed or endorsed by the publisher.
