# Supplementary material for: In vivo modelling of cutaneous T-cell lymphoma: The role of SOCS1
Source: Front Oncol. 2022 Nov 24;12:1031052. doi: 10.3389/fonc.2022.1031052 (PMC9730277; doi:10.3389/fonc.2022.1031052)
Supplement: Supplementary file 1 [file DataSheet_1.pdf]

Supplementary Material

1 Supplementary Figures and Tables

Table S1. List of the primer sequences used for genomic PCR

Forward (F) and reverse (R) primers are indicated.

Primer	Sequence
Socs1wt_F	5’- GCATCCCTCTTAACCCGGTAC-3’
Socs1wt_R	5’- AAATGAAGCCAGAGACCCTCC-3’
Socs1flox_F	5’- TTAGGCACTTGCTTCTGGTGC-3’
Socs1flox_R	5’- TTCTGGAAAGCTAGCACCACG-3’
Cd4CreER_F	5’- TACGGCGCTAAGGATGACTCT-3’
Cd4CreER_R	5’- ATCATGTGAACCAGCTCCCTG-3’

1.1 Supplementary Figures

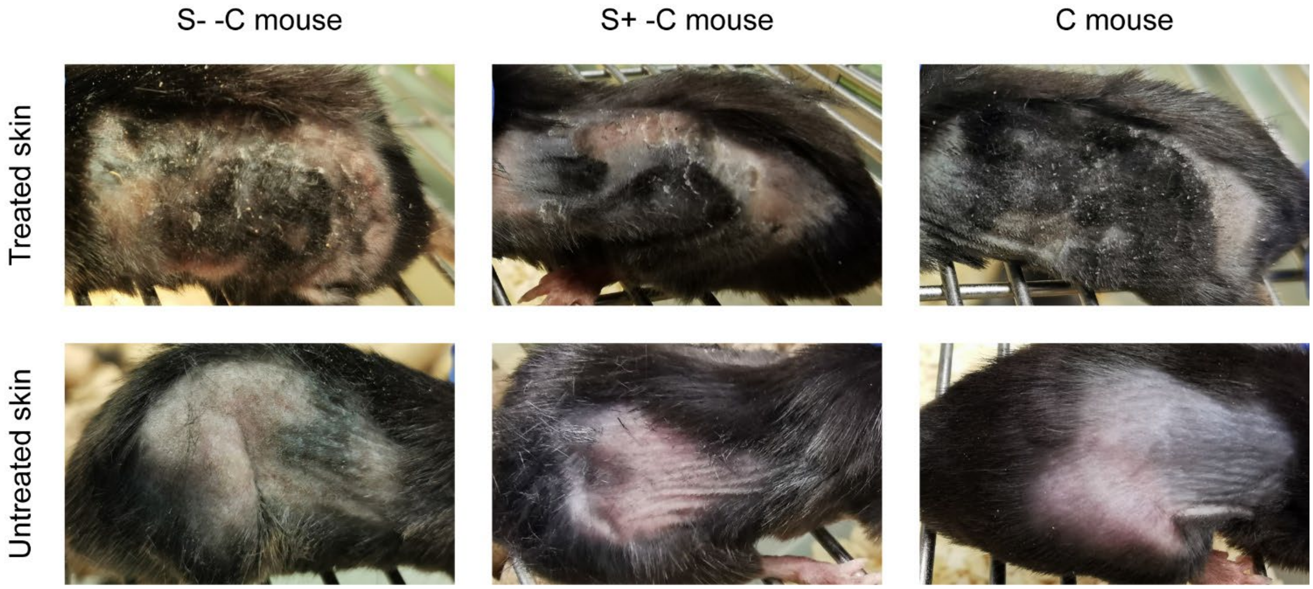

Supplementary Figure 1. Augmented skin inflammation induced by repeated low concentration oxazolone. Representative images of the shave treated skin and untreated skin of S--C mice, S+-C mice and C mice on day 35 during experiment.

S--C is Socs1 -/- Cd4Cre+/- ; S+-C is Socs1-/wt Cd4Cre+/-; C is Socs1wt/wt Cd4Cre+/-.
